# Minimizing Contrast Use in High-Risk Patients: A Case Report of Optical Coherence Tomography-Guided Percutaneous Coronary Intervention

**DOI:** 10.7759/cureus.105185

**Published:** 2026-03-13

**Authors:** Aditi Newaskar, Raja Godasi, Ankush Gupta, Krishna Prasad

**Affiliations:** 1 Cardiology, All India Institute of Medical Sciences, Mangalagiri, IND; 2 Neurology, St. Luke's Boise Medical Center, Boise, USA; 3 Cardiology, Military Hospital, Jaipur, IND; 4 Cardiology, NRI Medical College, Vijayawada, IND

**Keywords:** complex pci, contrast-induced nephropathy, intravascular ultrasonography, optical coherence tomography (oct), type 1 myocardial infarction

## Abstract

Contrast-induced nephropathy (CIN) is a concerning complication following percutaneous coronary intervention (PCI), particularly in patients with preexisting renal impairment, diabetes, hypertension, and heart failure. Intravascular imaging with optical coherence tomography (OCT) requires contrast flushes for clearing blood and can cause CIN. We present a patient at high risk for developing CIN who underwent PCI with minimal contrast use, aided by OCT with saline flushes and a parallel wire technique to visualize plaque morphology and guide stent placement, while avoiding excessive contrast exposure. The patient had no worsening of kidney function after the procedure. Despite the absence of intravascular ultrasound, the parallel wire technique facilitated accurate stent positioning, contributing to a successful PCI outcome. This case underscores the importance of minimizing contrast use in high-risk patients and highlights the efficacy of OCT with saline flushes as an alternative imaging modality in reducing the risk of CIN during PCI.

## Introduction

Contrast-induced nephropathy (CIN) is a concerning complication following percutaneous coronary intervention (PCI), particularly in patients with preexisting renal impairment. CIN increases morbidity and prolongs hospital stays, adding to costs involved [1]. Optical coherence tomography (OCT), an innovative intravascular imaging technique, enhances the identification of plaque morphology and evaluates pre- and postintervention characteristics, such as plaque morphology, landing zones for stent deployment, and stent optimization [2]. Numerous randomized controlled trials have demonstrated that OCT can reduce major adverse cardiovascular events. However, OCT imaging necessitates the use of contrast to clear blood from the imaging field, posing a challenge for patients at high risk of developing CIN [3]. Consequently, these high-risk patients are typically excluded from major trials. In these patients, saline flushes provide an effective alternative to contrast, allowing for adequate imaging without the associated risks. Minimizing the volume of contrast is one of the effective strategies in decreasing the risk of CIN post-PCI. Intravascular ultrasound (IVUS) is the preferred imaging method for minimizing contrast use in these cases [4]. In this report, we described a patient with a high risk of developing CIN, who underwent PCI with minimal contrast use, aided by OCT and a parallel wire technique. Despite the absence of IVUS, the parallel wire technique facilitated accurate stent positioning, contributing to a successful PCI outcome.

## Case presentation

**Figure 1:**
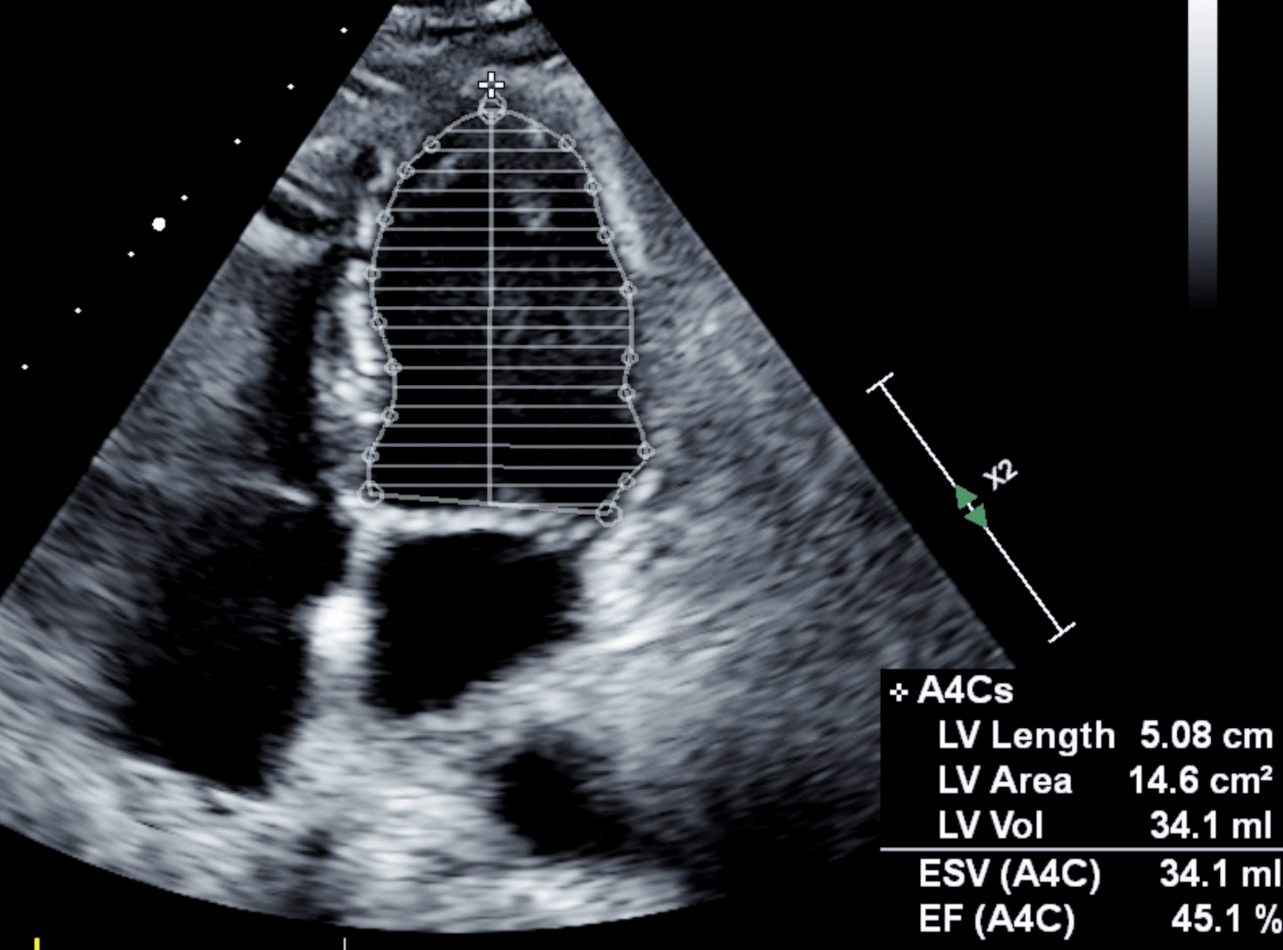
Echocardiogram in the apical four-chamber view, showing an ejection fraction of 45% A4C: apical four-chamber view; LV: left ventricle; ESV: end-systolic volume; EF: ejection fraction

A 74-year-old male diabetic, hypertensive, and chronic kidney disease patient presented with chest pain and was diagnosed with non-ST-segment elevation myocardial infarction. Baseline creatinine was 2.3 mg/dL (normal range 0.7 - 1.3 mg/dL). His echocardiogram (ECG) showed an ejection fraction of 45% with hypokinesia in the left anterior descending artery (LAD) territory (basal and mid anterior and anteroseptal segments) (Figure 1).

**Figure 2:**
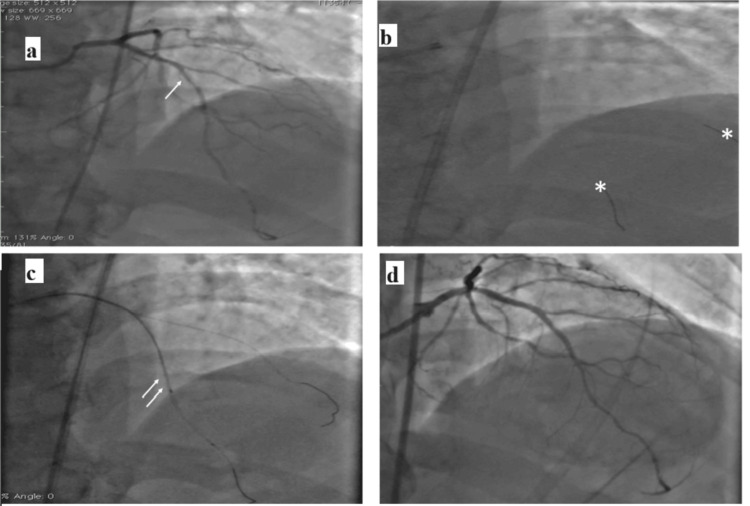
(a) Angiogram showing significant stenosis in proximal to the mid-left anterior descending artery (arrow). (b) Work horse wires placed in the left anterior descending artery and major diagonal across the lesion (asterisks). (c) Angiogram image showing the positioning of optical coherence tomography catheter (double arrows). (d) Final angiogram showing well-apposed stent LAD: left anterior descedning artery

Post-angiogram performed with minimal contrast showed significant mid-LAD stenosis (Figure 2a, Video 1).

**Video 1:** Coronary angiogram in the anteroposterior cranial view showing significant stenosis in the mid left anterior descending artery

**Figure 3:**
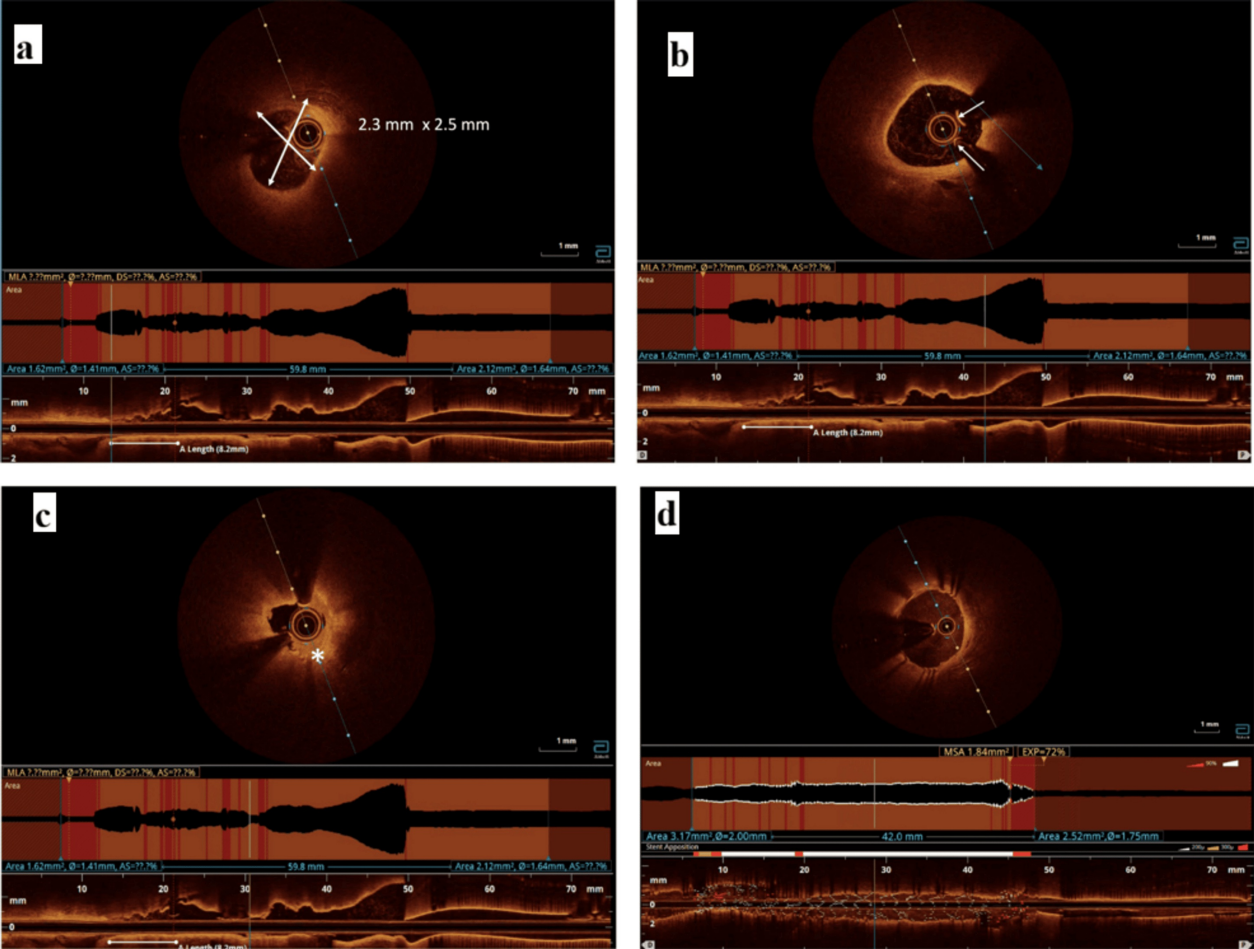
(a) Optical coherence tomography image showing the distal landing zone with reference diameters. (b) Proximal landing zone (note made of two wire artifacts (arrows)). (c) Minimal luminal area segment showing a fibrous plaque (asterisk). (d) Post-stent deployment optical coherence tomography image showing well-expanded and apposed stent

Given the high risk of CIN, minimal-contrast PCI was planned. As IVUS was unavailable, we planned OCT with saline flushes. Extra Back-Up 3.5 6-Fr catheter (Medtronic, Minneapolis, MN) was used to cannulate the left main artery, with engagement confirmed by injecting saline and ECG monitoring. Then, two floppy wires were passed in LAD and a large diagonal with angiographic images as reference (Figure 2b). These multiple wires helped us silhouette the LAD. Then, OCT runs were taken with a 20 mL saline flush and hand injection. OCT showed a fibrous plaque (Figures 2c, 3c and Video 2). Distal (2.38 mm, located 18 mm from the diagonal ostium) and proximal (3.0 mm, located 20 mm from the diagonal ostium) landing zones were identified (Figures 3a, 3b).

**Video 2:** Prestenting optical coherence tomography images with saline flushes

Based on these measurements, a 2.75 x 40 mm drug-eluting stent was selected. The stent is deployed under fluoroscopic guidance, with half of the stent beyond the diagonal wire. It is assumed that, once the distal landing zone is reached, the proximal landing zone will also be reached. Postdeployment OCT showed minor stent malapposition with good expansion (Figure 3d, Video 3), which was optimized with noncompliant balloon dilatation. Final angiogram with 7 mL contrast showed no wire exit perforations and Thrombolysis in Myocardial Infarction III flow (Figure 2d, Video 4).

**Video 3:** Poststenting optical coherence tomography video showing well-apposed and well expanded stents with no edge dissections

**Video 4:** Final angiogram showing well-apposed stent and no wire exit perforations

Postprocedure renal function tests remained stable, and the patient was asymptomatic at six-month follow-up.

## Discussion

We have demonstrated, in this case, how to use OCT with saline flushes for minimal contrast PCI by silhouetting the target vessel with additional wires to guide stent placement. We hardly used any contrast except for the final injection to look for any wire exit perforation. This technique helped us in preventing CIN.

CIN post-PCI is detrimental, leading to morbidity, mortality, increased length of hospital stay, and financial burden. Contrast use should be minimal in those patients who are at high risk of developing CIN. Any dynamic change in renal function during acute coronary syndrome (ACS) is associated with poor long-term outcomes. Even transient acute kidney injury (AKI) during ACS is associated with increased long-term mortality, compared with patients without AKI, with an adjusted hazard ratio of 1.2 [1].

Intravascular imaging, such as OCT and IVUS, has led to a reduction in major adverse cardiac events [2]. The use of OCT needs higher contrast volume, which has led to a higher incidence of CIN after OCT in various studies. IVUS has been used for minimal-contrast studies and can be performed easily because the pullback can be stopped at any point, and that point can be referenced to the nearest bony landmarks [3,4]. However, in OCT cases, the pullback cannot be interrupted.

OCT requires contrast flushes to displace blood; however, studies suggest saline and dextran can be used as alternative media [3,5]. Although OCT with zero or minimal contrast has been performed in a few reports, it was primarily performed with angio co-registration (ACR) [6]. Therefore, in cases where ACR was not available, the parallel wire technique for silhouetting the main vessel can help in stent positioning. This does not require additional contrast use and, thus, decreases the risk of CIN. This is the first time multiple wires were used to silhouette the vessel and were used as a guide for stent positioning.

## Conclusions

This case highlights the judicious use of contrast in preventing CIN. CIN is a preventable complication, and every effort should be made to avoid it. Intravascular imaging like OCT helps in optimizing stent deployment, albeit with increased risk of AKI. Using saline flushes as an alternative to contrast is effective and provides interpretable OCT images, thereby minimizing the risk of CIN. Using a parallel wire to silhouette the vessel allows us to minimize contrast use, thereby reducing the incidence of CIN.
